# Combined paclitaxel and gemcitabine as first-line treatment in metastatic non-small cell lung cancer: a multicentre phase II study

**DOI:** 10.1054/bjoc.2001.1784

**Published:** 2001-05

**Authors:** J Y Douillard, D Lerouge, A Monnier, J Bennouna, A M Haller, X S Sun, D Assouline, B Grau, A Rivière

**Affiliations:** Centre René Gauducheau, 44805 Saint-Herblain, France; Centre François Baclesse, 14076 Caen, France; C.H.G André Boulloche, 25209 Montbeliard, France; C.H.U. de Nancy Hôpital de Brabois, 54511 Vandoeuvre, France; Clinique du Mail, 38034 Grenoble, France; Bristol-Myers Squibb, 92044 Paris la Défense, France

**Keywords:** paclitaxel, gemcitabine, chemotherapy, non-small cell lung cancer

## Abstract

The efficacy and toxicity of combined paclitaxel and gemcitabine was evaluated in 54 chemotherapy-naive patients with metastatic non-small cell lung cancer (NSCLC). Gemcitabine i.v. 1000 mg/m^2^was administered on days 1 and 8 and paclitaxel 200 mg/m^2^as a continuous 3-hour infusion on day 1. Treatment was repeated every 21 days. Patients had a median age of 53 years. ECOG performance status was 0 or 1 in 48 patients. 41 patients (75.9%) had initial stage IV disease; histology was mainly adenocarcinoma (46.3%). 2 patients (4.3%) achieved a complete response and 15 (31.9%) achieved a partial response giving an overall response rate of 36.2% (95% CI: 22.4–49.9%); 19 patients (40.4%) had stable disease and 10 (21.3%) had progressive disease. The median survival time was 51 weeks (95% CI: 46.5–59.3), with a 1-year survival probability of 0.48 (95% CI: 0.34–0.63). Grade 3/4 neutropenia and febrile neutropenia occurred in 15.2% and 2.2% of courses, respectively. Grade 3/4 thrombocytopenia was rare (1.8% of courses). Peripheral neurotoxicity developed in 25 patients (47.2%), mostly grade 1/2. Arthalgia/myalgia was observed in 30 patients (56.6%), generally grade 1 or 2. Grade 3 abnormal levels of serum glutamate pyruvate transaminase (SGPT) and serum glutamate oxaloacetate transaminase (SGOT) occurred in 5 patients (9.4%) and 1 patient (1.9%), respectively. Combined paclitaxel and gemcitabine is an active and well-tolerated regimen for the treatment of advanced NSCLC, and warrants further investigation in comparative, randomized trials. © 2001 Cancer Research Campaign http://www.bjcancer.com


*British Journal of Cancer* (2001) **84**(9), 1179–1184
© 2001 Cancer Research Campaign
doi: 10.1054/ bjoc.2001.1784, available online at http://www.idealibrary.com on IDEAL®

http://www.bjcancer.com

### Combined paclitaxel and gemcitabine as first-line treatment in metastatic non-small cell lung cancer: a multicentre phase II study

**JY Douillard[1], D Lerouge[2], A Monnier[3], J Bennouna[1], AM Haller[4], XS Sun[3], D Assouline[5], B Grau[6] and A Rivière[2]**

[1]Centre René Gauducheau, 44805 Saint-Herblain, France; [2]Centre François Baclesse, 14076 Caen, France; [3]C.H.G André Boulloche, 25209 Montbeliard, France; [4]C.H.U. de Nancy Hôpital de Brabois, 54511 Vandoeuvre, France; [5]Clinique du Mail, 38034 Grenoble, France; [6]Bristol-Myers Squibb, 92044 Paris la Défense, France

**Summary** The efficacy and toxicity of combined paclitaxel and gemcitabine was evaluated in 54 chemotherapy-naive patients with metastatic non-small cell lung cancer (NSCLC). Gemcitabine i.v. 1000 mg/m$^2$ was administered on days 1 and 8 and paclitaxel 200 mg/m$^2$ as a continuous 3-hour infusion on day 1. Treatment was repeated every 21 days. Patients had a median age of 53 years. ECOG performance status was 0 or 1 in 48 patients. 41 patients (75.9%) had initial stage IV disease; histology was mainly adenocarcinoma (46.3%). 2 patients (4.3%) achieved a complete response and 15 (31.9%) achieved a partial response giving an overall response rate of 36.2% (95% CI: 22.4–49.9%); 19 patients (40.4%) had stable disease and 10 (21.3%) had progressive disease. The median survival time was 51 weeks (95% CI: 46.5–59.3), with a 1-year survival probability of 0.48 (95% CI: 0.34–0.63). Grade 3/4 neutropenia and febrile neutropenia occurred in 15.2% and 2.2% of courses, respectively. Grade 3/4 thrombocytopenia was rare (1.8% of courses). Peripheral neurotoxicity developed in 25 patients (47.2%), mostly grade 1/2. Arthalgia/myalgia was observed in 30 patients (56.6%), generally grade 1 or 2. Grade 3 abnormal levels of serum glutamate pyruvate transaminase (SGPT) and serum glutamate oxaloacetate transaminase (SGOT) occurred in 5 patients (9.4%) and 1 patient (1.9%), respectively. Combined paclitaxel and gemcitabine is an active and well-tolerated regimen for the treatment of advanced NSCLC, and warrants further investigation in comparative, randomized trials. © 2001 Cancer Research Campaign http://www.bjcancer.com

**Keywords**: paclitaxel; gemcitabine; chemotherapy; non-small cell lung cancer

Recently, paclitaxel and then gemcitabine have emerged as promising new agents in first-line treatment of locally advanced and metastatic non-small cell lung cancer (NSCLC). In several phase II trials, single-agent paclitaxel has produced response rates of 21–38% and a 1-year survival rate of 35–42% (Chang et al, 1993; Murphy et al, 1993; Alberola et al, 1995; Gatzemeier et al, 1995), while response rates of 20–26% have been reported for single-agent gemcitabine, with 1-year survival rates of 31–43% (Anderson et al, 1994; Gatzemeier et al, 1996; Fukuoka et al, 1997; Yokoyama et al, 1997).

Gemcitabine is a novel deoxycytidine analogue, which acts as a competitive substrate for incorporation into DNA where it leads to termination of DNA chain elongation (Plunkett et al, 1995). In contrast, paclitaxel has no direct action on DNA synthesis, but acts by promoting the polymerization of tubulin into stable microtubules and inhibiting the formation of stable microtubule bundles, ultimately leading to cell death (Schiff et al, 1979). Common adverse events of gemcitabine are myelosuppression (mainly neutropenia), hepatic abnormalities and nausea/vomiting (Noble and Goa, 1997), while those commonly associated with paclitaxel include neutropenia, anaemia, peripheral neuropathy, myalgia/arthalgia, mucositis and alopecia (Wiseman and Spencer, 1998). Based on their single-agent activity, different mechanisms of action and essentially non-overlapping toxicities (Rowinsky and Donehower 1995; Peters and Ackland, 1996), it seems important to explore the potential of paclitaxel and gemcitabine in combination. This approach is supported by the lack of pharmacokinetic interaction between the two drugs and the ability of paclitaxel to increase cellular accumulation of gemcitabine triphosphate (dFdCTP), the active metabolite of gemcitabine, with the possibility of enhancing its antitumor activity (Kroep et al, 1999). Several phase I studies involving different schedules of paclitaxel and gemcitabine in a variety of tumour types have been encouraging and mainly found neutropenia and elevated transaminase levels to be dose-limiting (Poole et al, 1997; Sandler et al, 1997). In a phase I/II dose-finding study in advanced NSCLC, gemcitabine 1000 mg/m$^2$ was administered on days 1 and 8, and paclitaxel 150 to 200 mg/m$^2$ as a 3-hour infusion on day 1 of a 21-day cycle (Giaccone et al, 1998a). Preliminary data revealed that among the first 30 patients enrolled, dose escalation was well tolerated and the response rate was 30%; any failure to undergo dose escalation was mainly unrelated to adverse effects. This schedule was also investigated in a phase I/II study of the pharmacokinetic and pharmacodynamic interactions between gemcitabine and paclitaxel in patients with NSCLC (Kroep et al, 1999). Due to mild toxicity, the dose of paclitaxel was increased from 150 to 200 mg/m$^2$.

Consequently, in view of the relatively mild toxicity profile, the possibility of improved patient outcome, and the advantage of dose administration on an out-patient basis, the present phase II study was designed to investigate further the efficacy and safety profile of combined paclitaxel and gemcitabine in advanced NSCLC.

*Received 2 October 2000*
*Revised 14 February 2001*
*Accepted 20 February 2001*

*Correspondence to:* JY Douillard

### PATIENTS AND METHODS

### Patient population

Chemotherapy-naive patients with histologically or cytologically confirmed stage IV (Mountain, 1997) or relapsed metastatic NSCLC after surgery and/or radiotherapy were included in the study. Further inclusion criteria were: age between 18 and 75 years; Eastern Cooperative Oncology Group (ECOG) performance status ≤ 2 (Minna et al, 1984); life expectancy ≥12weeks; at least one bidimensionally measurable lesion (2 cm × 2 cm minimum) located outside previously irradiated locations; and adequate haematological (absolute neutrophil count [ANC] ≥1 500 $\mu l^{-1}$, platelet count ≥100 000 $\mu l)^{-1}$, renal (serum creatinine ≤1.5 × upper normal limit), and hepatic (bilirubin ≤1.5 × upper normal limit, serum glutamate oxaloacetate transaminase (SGOT) and serum glutamate pyruvate transaminase (SGPT) ≤2.5 × upper normal limit) functions. Patients were excluded if they had brain metastasis, a history of neoplasm (except cured non-melanoma skin carcinoma or carcinoma in-situ of the cervix), history of cardiac disease (uncontrolled hypertension, unstable angina, congestive heart failure, second- or third-degree heart block, myocardial infarction within the previous year, cardiac ventricular arrhythmias requiring medication), peripheral neuropathy, a psychiatric disorder, serious active infection, or allergic reaction to preparations containing cremophor. Females of childbearing potential had to have a negative serum or urine pregnancy test within 48 hours of enrolment and had to use adequate contraceptive measures during the study. Pregnant or lactating women were excluded. Patients with previous radiotherapy were included providing treatment was completed 4 weeks before starting treatment and they had recovered from all adverse effects, and less than 30% of marrow-bearing bones were irradiated. Also, major surgery must have been completed at least 2 weeks before enrolment. Approval of the study (including the informed consent form) was given by the Consultative Committee for the Protection of Persons involved in Biomedical Research of Nantes, and all patients gave written informed consent.

### Patient evaluation

Pretreatment evaluation included a physical examination, electrocardiogram (ECG), and laboratory tests (haematology and biochemistry). Tumour sites were assessed by physical examination and computed tomography (CT) scans of the thorax, abdomen and brain. An isotopic bone scan or X-ray was taken to detect bone metastases and assess as much as possible the disease extension, a known prognostic factor. During treatment, a physical examination, a pregnancy test (if applicable), an ECG, and haematology and biochemistry assessments preceded each treatment course. Before the second dose of gemcitabine on every treatment course (day 8), patients had an ECG and haematology tests (haemoglobin, white blood cells, ANC and platelets count).

Tumour sites were evaluated by physical examination every cycle and by CT imaging every 2 cycles. Adverse events were evaluated according to The National Cancer Institute Common Toxicity Criteria (NCI-CTC) scale. On study completion or discontinuation, follow-up of disease status, survival and tolerance was performed every 3 months until disease progression. After progression, follow-up for survival continued every 3 months for the first 2 years and then every 6 months.

### Treatment schedule

Gemcitabine (Gemzar®; Eli-Lilly, Indianapolis, IN) 1000 mg/m$^2$ was administered as a 30 minute intravenous infusion on day 1 and day 8. On day 1 gemcitabine was given before paclitaxel (Taxol®; Bristol-Myers Squibb, Mayaguez, Puerto Rico) 200 mg/m$^2$ diluted in 500 ml of 5% dextrose (final concentration was not to exceed 1.2 mg ml)$^{-1}$ administered as a 3 hour infusion. Premedication to prevent possible anaphylactic reaction comprised intravenous dexamethasone 20 mg, dexchlorpheniramine 5 mg, and cimetidine 300 mg or ranitidine 50 mg, all given 30 minutes before paclitaxel. Courses were repeated every 21 days or upon haematologic recovery (ANC ≥1500 $\mu l^{-1}$ and platelet count ≥100 000 $\mu l)^{-1}$. If haematologic recovery was not achieved by day 35, treatment was discontinued. Gemcitabine administration on day 8 could be delayed to day 15 according to haematologic recovery (ANC ≥ 1000 $\mu l^{-1}$ and platelet count ≥100 000 $\mu l^{-1}$).

Dose reductions to paclitaxel 175 mg/m$^2$ and gemcitabine 750 mg/m$^2$ were made in case of haematologic toxicity (ANC <500 $\mu l^{-1}$ for ≥7 days, febrile neutropenia, grade 4 thrombocytopenia, grade 4 anaemia, bleeding episode requiring platelet transfusion) and nonhaematologic toxicity (mucositis with ulcers WHO grade ≥3). Doses were reduced to paclitaxel 150 mg/m$^2$ and gemcitabine 500 mg/m$^2$ for elevated bilirubin levels grade 3. Dose reductions in paclitaxel alone to 175 mg/m$^2$ were made for severe myalgia/arthralgia or peripheral neurotoxicity grade 2 (a further reduction to paclitaxel 150 mg/m$^2$ was made if peripheral neurotoxicity grade 2 persisted). Dose re-escalation was not allowed. Treatment was discontinued in case of severe myalgia/arthralgia lasting ≥7 days, hepatotoxicity (bilirubin grade 4, persistent elevated transaminases grade 3 or 4), peripheral neurotoxicity grade 3, symptomatic arrhythmia or heart block (except first degree AV block), or other major organ toxicity grade 3 or 4 (except alopecia or vomiting) not recovered after dose reduction or a 2-week delay. Other anticancer drugs, immunotherapy and radiotherapy were prohibited. Treatment was continued in the absence of disease progression and unacceptable toxicity for a maximum of 10 courses.

### Criteria for response

Response to treatment was assessed every two courses according to World Health Organization (WHO) response criteria (Miller et al, 1981). Complete response (CR) required disappearance of all clinical evidence of tumour, determined by 2 observations at least 4 weeks apart. Partial response (PR) required 50% or more reduction in the sum of the products of the perpendicular dimensions of measured lesions, determined by 2 observations at least 4 weeks apart without the appearance of new lesions. Stable disease (SD) was defined as a decrease in lesion size of less than 50% in the sum of the products of measured lesions or progression less than 25% for a minimum of 4 weeks, without the appearance of new lesions. Progressive disease (PD) was defined as an increase in lesion size of at least 25% or the appearance of new lesions. Bone disease was evaluated separately in the reporting of CR and patients with bone metastases were included in the reporting of overall response (CR and PR) according to a separate set of response criteria (CR was complete disappearance of all lesions on X-ray or scan for at least 4 weeks, without the appearance of new lesions; PR was at least a 50% decrease in the size of lytic lesions, or decreased density of blastic lesions for at least 4 weeks); SD was not applied until at least 8 weeks after the start of therapy

 *© 2001 Cancer Research Campaign*

because of the slow response of bone lesions; PD was an increase in size of existing lesions or appearance of new lesions.

### Statistics

All patients who received at least 2 courses of treatment were evaluable for response. In case of progression following the first course, patients were evaluated as 'early progression'. Patients who received at least one course of treatment were assessable for toxicity. The study used a 2-stage Simon optimum design (Simon, 1989), where a population response rate of less than 15% is considered insufficiently effective and one of 35% or more is considered worthy of further investigation. In the first stage of the study, 19 patients evaluable for response were considered and if 4 or more patients showed a partial or complete response, a further 25 response-evaluable patients were enrolled. If 11 of 44 patients responded, the treatment regimen was considered a useful combination for phase III studies. This procedure has a power of 90% to detect a true response rate of 35%, at a confidence level of 5%. A two-sided exact 95% confidence test was performed on the response rate (ratio between the number of patients with complete or partial response and the total number of patients studied). The Kaplan–Meier method (Kaplan and Meier, 1958) was used to calculate time to response (time from enrolment to complete or partial response), response duration (time from partial or complete response to disease progression), progression-free survival (time from enrolment to disease progression), and overall survival (time from enrolment to death).

### RESULTS

### Patient characteristics

A total of 54 patients (49 men and 5 women), with a median age of 53 years (range, 37–74 years) were enrolled in the study. Most patients had a performance status of 0–1 (88.9%; one patient had a performance status of 3 that exceeded the inclusion criteria, this patient was excluded from the efficacy and safety evaluation), initial stage IV disease (75.9%), and adenocarcinoma (46.3%) (Table 1). 53 patients were assessable for toxicity, and 47 fulfilled all criteria for response evaluation.

**Table 1** Patient characteristics

| | | | Patients | |
|---|---|---|---|---|
| | | | Number | % |
| Total | | | 54 | |
| Sex | | | | |
| Male | | | 49 | 90.7% |
| Female | | | 5 | 9.3% |
| Age (years) | | | | |
| Median | | 53 | | |
| Range | | 37–74 | | |
| ECOG performance status | | | | |
| 0 | | | 15 | 27.8 |
| 1 | | | 33 | 61.1 |
| 2 | | | 5 | 9.3 |
| 3 | | | 1 | 1.9 |
| Histology | | | | |
| Adenocarcinoma | | | 25 | 46.3 |
| Squamous cell carcinoma | | | 15 | 27.8 |
| Large-cell carcinoma | | | 10 | 18.5 |
| Other | | | 4 | 7.4 |
| Stage | | | | |
| IIIB (lymphangitis or pleural effusion) | | | 5 | 9.3 |
| IV | | | 41 | 75.9 |
| Metastatic relapse | | | 8 | 14.8 |
| Metastatic sites: | bone | | 14 | |
| | adrenal | | 10 | |
| | liver | | 14 | |
| | lung | | 5 | |
| | brain | | 1 | |
| | kidney | | 2 | |
| | abdominal nodes | | 10 | |
| Prior therapy | | | | |
| Radiotherapy | | | 13 | 24.1 |
| Surgical resection | | | 9 | 16.7 |

### Compliance with treatment

A total of 276 courses were administered to 53 patients, with a median of 6 courses per patient (range, 1–10) and the median interval between courses was 21 days (range, 20–35 days). Mean dose intensities for paclitaxel and gemcitabine were 65 mg/m$^2$/week (97.6%) and 659 mg/m$^2$/week (94.3%) respectively. 30 patients (56.6%) received 6 or more courses and 3 (5.7%) received all 10 courses. Treatment was delayed in 23 courses (8.3%), for haematologic complications in 5 courses (3 patients, 5.7%) and nonhaematologic complications in 2 courses (1 patient, 1.9%), and other reasons, mainly non-medical, in 16 courses (14 patients, 26.4%). The scheduled dose of gemcitabine on day 8 was omitted completely in 5 courses (5 patients, 9.4%) due to left ventricular decompensation, dyspnoea and pneumothorax, and haematologic toxicity in one course, and disease progression in two courses. Three, day-8 doses of gemcitabine were delayed until day 15 in one patient, because of haematologic toxicity in two courses and by error in one course. One infusion of paclitaxel was temporarily interrupted by a hypersensitivity reaction. Paclitaxel dose reductions were required in 7 courses (7 patients, 13.2%) due to nonhaematologic toxicity (peripheral neurotoxicity in 4, arthromyalgia in 3). No dose reduction was required for gemcitabine alone. Both paclitaxel and gemcitabine doses were reduced in 3 courses due to haematologic toxicity in 2 (thrombocytopenia grade 4 and febrile neutropenia grade 4) and nonhaematologic toxicity in 1 (transient increase in SGPT and SGOT).

### Toxicity

The major haematologic and nonhaematologic toxicities associated with this regimen are shown in Tables 2 and 3. Grade 3 and 4 neutropenia occurred in 15.2% of treatment courses, and febrile neutropenia was observed in 2.2% of courses. Episodes of grade 3 and 4 thrombocytopenia were infrequent, occurring in 1.8% of

**Table 2** Haematologic toxicity, in all treatment courses ($n$ = 276)

| Toxicity | Number of treatment courses (%) | | | |
|---|---|---|---|---|
| | NCI-CTC[a] grade | | | |
| | 1 | 2 | 3 | 4 |
| Neutropenia | 30 (10.9) | 13 (4.7) | 29 (10.5) | 13 (4.7) |
| Thrombocytopenia | – | 4 (1.4) | 3 (1.1) | 2 (0.7) |
| Anaemia | – | 47 (17.0) | 6 (2.2) | – |
| Infection | 1 (0.4) | 1 (0.4) | – | – |

[a]The National Cancer Institute Common Toxicity Criteria.

© 2001 Cancer Research Campaign

**Table 3** Nonhaematologic toxicity, in 53 assessable patients

| Toxicity | Number of patients (%) | | | |
|---|---|---|---|---|
| | NCI-CTC[a] grade | | | |
| | 1 | 2 | 3 | 4 |
| Neurotoxicity | 10 (18.9) | 13 (24.5) | 2 (3.8) | – |
| Stomatitis | 6 (11.3) | 1 (1.9) | 1 (1.9) | – |
| Myalgia/arthralgia | 12 (22.6) | 14 (26.4) | 4 (7.5) | – |
| Nausea/vomiting | 14 (26.4) | 13 (24.5) | 2 (3.8) | – |
| Alopecia | 5 (9.4) | 43 (81.1) | – | – |
| Hypersensitivity reactions | 3 (5.7) | 1 (1.9) | 1 (1.9) | – |

[a]The National Cancer Institute Common Toxicity Criteria.

courses and were not complicated by haemorrhage. No grade 4 anaemia was observed. Nonhaematologic toxic effects were generally mild to moderate. Peripheral neurotoxicity was reported in 25 patients (47.2%), mostly grade 1 and 2 and symptoms were not more severe or more prolonged than may have been expected from paclitaxel alone. Arthalgia/myalgia was observed in 30 patients (56.6%), but episodes were generally grade 1 or 2. Grade 3 nausea/vomiting was reported in 2 patients (3.8%); no grade 4 toxicity occurred. Nephrotoxicity (abnormal levels of serum creatinine) did not exceed grade 1. Hepatotoxicity was mainly mild; grade 2 abnormal levels of total bilirubin were observed in 52 patients (98.1%). Grade 3 abnormal levels of SGPT and SGOT occurred in 5 patients (9.4%) and 1 patient (1.9%), respectively; no grade 4 levels were reported. Hypersensitivity reactions were observed in 5 patients (9.4%). Cardiotoxicity was reported in 4 patients (7.5%) as pericardial effusion and atrial flutter (1 patient), and either left ventricular failure, parasympatic malaise or thoracic pain (1 patient each). 7 patients (13.2%) discontinued treatment due to toxic effects (paraesthesia in 2; hepatotoxicity in 3; 1 each for myalgia grade 3 and cardiac failure).

### Response and survival

Among 47 assessable patients, 2 patients (4.3%) achieved a CR and 15 patients (31.9%) achieved a PR, giving an overall response rate of 36.2% (95% CI: 22.4–49.9%). 5 out of 15 patients who achieved PR had initial bone metastasis. 19 patients (40.4%) had stable disease and 10 (21.3%) had progressive disease. Determination of response was not possible in one patient (2.1%). The overall response rate was 32.1% (95% CI: 19.5–44.6%) for the population who received at least one course of treatment. The median time to response was 11.5 weeks (95% CI: 6–12). The median duration of response was 22.5 weeks (95% CI: 19–29) and the median duration of progression-free survival was 25 weeks (95% CI: 18–31.3). The median survival time was 51 weeks (95% CI: 46.5–59.3), with a 1-year survival probability of 0.48 (95% CI: 0.34–0.63) (Figure 1). Response was achieved in all histologic types, squamous cell (5 of 11 evaluable patients, 45.5%), adenocarcinoma (8 of 23 evaluable patients, 34.8%) and large cell carcinoma (2 of 9 evaluable patients, 22.2%).

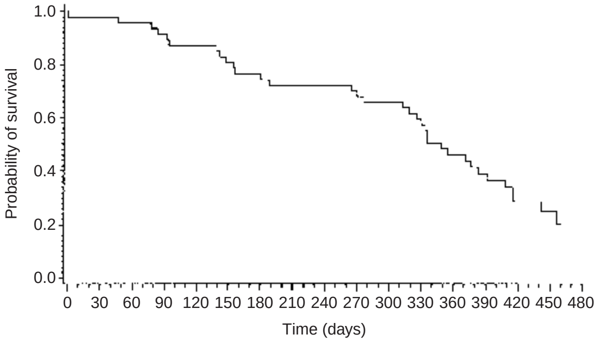

**Figure 1** Overall survival of patients with advanced NSCLC treated with combined paclitaxel and gemcitabine. The 1-year survival probability is 0.48 (95% CI: 0.34–0.63)

### DISCUSSION

Platinum-based combination chemotherapy has played a pivotal role in the treatment of advanced NSCLC. Examples of effective combinations include gemcitabine plus cisplatin (response rates of 30–54% and median survivals of 13–66 weeks (Abratt et al, 1997; Crino et al, 1997; Sandler et al, 2000)), paclitaxel plus cisplatin (response rates of 41–47% and an estimated median survival of 43 weeks (Klastersky and Sculier, 1995; Pirker et al, 1995; Giaccone et al, 1998b)), paclitaxel plus carboplatin (response rates from 27% to 62%, a median survival of 34.3–56.7 weeks and a 1-year survival of 32–54% depending on the dosing schedule of paclitaxel (Langer et al, 1995; Johnson et al, 1996; Hainsworth et al, 1998)), and navelbine plus cisplatin (a response rate of 43% and a median survival of 35.3 weeks (Depierre et al, 1994)).

However, the emergence of new agents with superior single-agent activity to cisplatin and carboplatin has presented an opportunity to investigate the efficacy and safety of non-platinum-containing combinations in this clinical setting (Lilenbaum and Green, 1993). Exploration of combined paclitaxel and gemcitabine in advanced NSCLC is particularly promising because of their confirmed activity as single-agents and predominantly nonoverlapping toxicities. The results of the present study (overall objective response rate of 36.2%, a median survival of 51 weeks, and a 1-year survival of 48%) indicate that combined paclitaxel and gemcitabine provides similar anticancer activity as the new platinum-based regimens for the first-line treatment of advanced NSCLC. Similar results (response rate of 37.5%, a median survival of 55.7 weeks and an actuarial 1-year survival of 50.7%) have been obtained in a recent phase II study of gemcitabine combined with docetaxel, another taxane (Georgoulias et al, 1999).

The most encouraging aspect of the present study was the acceptable safety profile obtained without the use of haematopoietic growth factors. Although grade 3 or 4 neutropenia occurred in 15.2% of cycles, febrile neutropenia developed in only 2.2% of courses and was easily managed. Episodes of grade 3 and 4 thrombocytopenia were infrequent, occurring in 1.8% of courses, but were not complicated by haemorrhage. There were no grade 4 nonhaematologic toxicities; peripheral neurotoxicity and arthralgia/myalgia (which occurred in 47.2% and 56.6% of patients, respectively) were mainly grade 1 or 2. The safety profile of paclitaxel-gemcitabine in the present study was clearly distinguishable from the safety profiles of 4 platinum-containing regimen reported in a recent randomized phase III trial (Schiller et al, 2000). Grade 4 neutropenia and Grade 3–4 febrile neutropenia occurred in 55%, 37%, 49% and 42%, and 16%, 4% 10% and 3% of patients in the gemcitabine-cisplatin, docetaxel-cisplatin, paclitaxel-carboplatin and paclitaxel-cisplatin groups, respectively.

Triplet regimens of paclitaxel and gemcitabine in combination with platinum compounds (cisplatin or carboplatin) have also been evaluated. At various dosing schedules, the response rates have

 *© 2001 Cancer Research Campaign*

ranged from 44% to 57% with 1-year survival rates of 42% to 45% (Frasci et al, 1999; Hainsworth et al, 1999; Sørensen et al, 1999) Myelosuppression was the commonest toxicity. From these studies, it is apparent that despite improvements in response, there was no survival advantage and the significant myelotoxicity suggests that paclitaxel/gemcitabine/cisplatin or carboplatin combinations may be more appropriate for patients with good performance status, possibly in a neoadjuvant setting.

In view of the favourable safety profile of combined paclitaxel and gemcitabine, coupled with encouraging response and survival rates, further comparative randomized trials are justified to analyse the quality-of-life and cost-effectiveness of this highly effective combination, in addition to defining any safety advantages over platinum-based regimens in advanced NSCLC.

### REFERENCES

Abratt RP, Bezwoda WR, Goedhals L and Hacking DJ (1997) Weekly gemcitabine with monthly cisplatin: effective chemotherapy for advanced non-small cell lung cancer. *J Clin Oncol* **15**: 744–749

Alberola V, Rosell R, Gonzalez-Larriba J-L, Molina F, Ayala F, Garcia-Conde J, Benito D and Perez JM (1995) Single-agent Taxol, 3-hour infusion in untreated advanced non-small lung cancer. *Ann Oncol* **6**(3): 49–52

Anderson H, Lund B, Bach F, Thatcher N, Walling J and Hansen HH (1994) Single agent activity of weekly gemcitabine in advanced non-small cell lung cancer: A phase II study. *J Clin Oncol* **12**: 1821–1826

Chang A, Kim K, Glick T, Anderson T, Karp D and Johnson D (1993) Phase II study of taxol, merbarone and piroxantrone in stage IV non-small cell lung cancer: The Eastern Cooperative Oncology Group Results. *J Natl Cancer Inst* **85**: 388–394

Crino L, Scagliotti G, Marangolo M, Figoli F, Clerici M, De Marinis F, Salvati F, Cruciani G, Dogliotti L, Pucci F, Paccagnella A, Adamo V, Altavilla G, Incoronata P, Trippetti M, Mosconi AM, Santucci A, Sorbolini S, Oliva C and Tonato M (1997) Cisplatin-gemcitabine combination in advanced non-small cell lung cancer (NSCLC). A phase II study. *J Clin Oncol* **15**: 297–303

Depierre A, Chastang C, Quoix E, Lebeau A, Blanchon F, Paillot N, Lemarie E, Milleron B, Moro D and Clavier J (1994) Vinorelbine plus cisplatin in advanced non-small cell lung cancer: a randomised trial. *Ann Oncol* **5**: 37–42

Frasci G, Panza N, Comella P, Nicolella GP, Natale M, Manzione L, Bilancia D, Cioffi R, Maiorino L, De Cataldis G, Belli M, Micillo E, Mascia V, Massidda B, Lorusso V, De Lena M, Carpagnano F, Contu A, Pusceddu G and Comella G (1999) Cisplatin, gemcitabine, and paclitaxel in locally advanced or metastatic non-small-cell lung cancer: A phase I-II study. *J Clin Oncol* **17**: 2316–2325

Fukuoka M, Takada M, Yokoyama A, Kurita Y and Niitani H (1997) Phase II studies of gemcitabine for non-small cell lung cancer in Japan. *Semin Oncol* **24** (2 Suppl 7): S7–42–S7–46

Gatzemeier U, Heckmayer M, Neuhauss R, Schluter I, Pawel JV, Wagner H and Dreps A (1995) Phase II study with paclitaxel for the treatment of advanced inoperable NSCLC. *Lung Cancer* **12** (suppl 2): 101–106

Gatzemeier U, Shepherd FA, Le Chevalier T, Weynants P, Cottier B, Groen HJ, Rosso R, Mattson K, Cortes-Funes H, Tonato M, Burkes RL, Gottfried M and Voi M (1996) Activity of gemcitabine in patients with non-small cell lung cancer: A multicenter extended phase II study. *Eur J Cancer* **32**: 243–248

Georgoulias V, Kouroussis C, Androulakis N, Kakolyris S, Dimopoulos MA, Papadakis E, Bouros D, Apostolopoulou F, Papadimitriou C, Agelidou A, Hatzakis K, Kalbakis K, Kotsakis A, Vardakis N and Vlachonicolis J (1999) Front-line treatment of advanced non-small-cell lung cancer with docetaxel and gemcitabine: A multicenter phase II trial. *J Clin Oncol* **17**: 914–920

Giaccone G, Smit E, Laan D, Splinter T, van Meerbeek J and Postmus P (1998a) Phase I/II study of paclitaxel and gemcitabine in advanced non-small cell lung cancer. *Proc Am soc Clin Oncol* **17**: 486a (abstr 1869)

Giaccone G, Splinter TAW, Debruyne C, Kho GS, Lianes P, van Zandwijk N, Pennucci MC, Scagliotti G, van Meerbeeck J, van Hoesel Q, Curran D, Sahmoud T, Postmus PE for the European Organization for Research and Treatment of Cancer Lung Cancer Cooperative Group (1998b) Randomized study of paclitaxel-cisplatin versus teniposide-cisplatin in patients with advanced non-small cell lung cancer. *J Clin Oncol* **16**: 2133–2141

Hainsworth JD, Urba WJ, Hon JK, Thompson KA, Stagg MP, Hopkins LG, Thomas M and Greco FA (1998) One-hour paclitaxel plus carboplatin in the treatment of advanced non-small-cell lung cancer: results of a multicentre, phase II trial. *Eur J Cancer* **34**: 654–658

Hainsworth JD, Burris HA, Erland JB, Morrissey LH, Meluch AA, Kalman LA, Hon JK, Scullin DC, Smith SW and Greco FA (1999) Phase I/II trial of paclitaxel by 1-hour infusion, carboplatin, and gemcitabine in the treatment of patients with advanced nonsmall cell lung cancer. *Cancer* **85**: 1269–1276

Johnson DH, Paul DM, Hande KR, Shyr Y, Blanke C, Murphy B, Lewis M and De Vore RF (1996) Paclitaxel plus carboplatin in advanced non-small-cell lung cancer: A phase II trial. *J Clin Oncol* **14**: 2054–2060

Kaplan EL and Meier P (1958) Non-parametric estimation from incomplete observations. *J Am Stat Assoc* **53**: 457–481

Klastersky J and Sculier JP (1995) Dose finding study of paclitaxel (Taxol) plus cisplatin in patients with non-small cell lung cancer. European Lung Cancer Working Party. *Lung Cancer* **12**(2): 117–125

Kroep JR, Giaccone G, Voorn DA, Smit EF, Beijnen JH, Rosing H, van Moorsel CJ, van Groeningen CJ, Postmus PE, Pinedo HM and Peters GJ (1999) Gemcitabine and paclitaxel: Pharmacokinetic and pharmacodynamic interactions in patients with non-small cell lung cancer. *J Clin Oncol* **17**: 2190–2197

Langer CJ, Leighton JC, Comis RL, O'Dwyer PJ, McAleer CA, Bonjo CA, Engstrom PF, Litwin S and Ozols RF (1995) Paclitaxel and carboplatin in combination in the treatment of advanced non-small-cell lung cancer: A phase II toxicity, response and survival analysis. *J Clin Oncol* **13**: 1860–1870

Lilenbaum RC and Green MR (1993) Novel chemotherapeutic agents in the treatment of non-small-cell lung cancer. *J Clin Oncol* **11**: 1391–1402

Miller AB, Hoogstraten B, Staquet M and Winkler A (1981) Reporting results of cancer treatment. *Cancer* **47**: 207–214

Minna JD, Higgins GA and Glatstein EJ (1984) Cancer of the lung. In Cancer: Principles and Practice of Oncology, DeVita V, Hellman S and Roxenburg S (eds) pp536. Lippincott: Philadelphia

Mountain CF (1997) Revisions in the international system for staging lung cancer. *Chest* (Jun) **111**(6): 1710–1717.

Murphy WK, Fossella FV, Winn RJ, Shin DM, Hynes HE, Gross HM, Davilla E, Leimert J, Dhingra H, Raber MN, Krakoff IH and Hong WK (1993) Phase II study of taxol in patients with untreated advanced non-small cell lung cancer. *J Natl Cancer Inst* **85**: 384–388

Noble S and Goa KL (1997) Gemcitabine A review of its pharmacology and clinical potential in non-small cell lung cancer and pancreatic cancer. *Drugs* **54**: 447–472

Peters GJ and Ackland SP (1996) New antimetabolites in preclinical and clinical development. *Exp Opin Investig Drugs* **5**: 637–679

Pirker R, Krajnik G, Zochbauer S, Malayeri R, Kneussl M and Huber H (1995) Paclitaxel/cisplatin in advanced non-small cell lung cancer (NSCLC). *Ann Oncol* **6**: 833–835

Plunkett W, Huang P, Xu YZ, Heinemann V, Grunewald R and Gandhi V (1995) Gemcitabine: Metabolism, mechanisms of action, and self-potentiation. *Semin Oncol* **22**: 3–10

Poole CJ, Perren T, Hogberg T, Cook J, Jenkins AH, Ridderheim M and Anderson K (1997) Phase I study to investigate alternate sequencing of the combination of gemcitabine and paclitaxel in ovarian carcinoma. *Eur J Cancer* **33** (8): S121 (abstr 543)

Rowinsky EK and Donehower RC (1995) Paclitaxel (Taxol). *N Engl J Med* **332**: 1004–1014

Sandler A, Raghavan D, Meropol N, Meyers T, Kindler H, Fox S, Perez R and Einhorn LH (1997) A phase I trial of gemcitabine plus paclitaxel combination therapy in patients with refractory solid tumors. *Eur J Cancer* **33** (8): S248 (abstr 1120)

Sandler AB, Nemunaitis J, Denham C, von Pawel J, Cormier Y, Gatzemeier U, Mattson K, Manegold C, Palmer MC, Gregor A, Nguyen B, Niyikiza C and Einhorn LH (2000) Phase III trial of gemcitabine plus cisplatin versus cisplatin alone in patients with locally advanced or metastatic non-small-cell lung cancer. *J Clin Oncol* **18**: 122–130

Schiff PB, Fant J and Horwitz SB (1979) Promotion of microtubule assembly in vitro by paclitaxel. *Nature* **277**: 665–667

Schiller JH, Harrington D, Sandler A, Belani C, Langer C, Krook J and Johnson DH, Eastern Cooperative Oncology Group (2000) A randomised phase III trial of four chemotherapy regimens in advanced non-small cell lung cancer (NSCLC). *Proc Am Soc Clin Oncol* **19**: 2 (abstr)

Simon R (1989) Optimal two-stage designs for phase II clinical trials. *Controlled Clinical Trials* **10**: 1–10

Sørensen JB, Stenbygaard LE, Dombernowsky P and Hansen HH (1999) Paclitaxel, gemcitabine, and cisplatin in non-resectable non-small-cell, lung cancer. *Ann Oncol* **10**: 1043–1049

*© 2001 Cancer Research Campaign*

Wiseman LR and Spencer CM (1998) Paclitaxel An update of its use in the treatment of metastatic breast cancer and ovarian and other gynaecological cancers. *Drugs & Aging* **12**: 305–334

Yokoyama A, Nakai Y, Yoneda S, Kurita Y and Niitani H (1997) Activity of gemcitabine in the treatment of patients with non-small cell lung cancer. A multicenter phase II study. *Anticancer Drugs* **8**: 574–581

*© 2001 Cancer Research Campaign*
